# Therapeutic potential of *Boswellia*-derived compounds in skin conditions

**DOI:** 10.3389/fphar.2026.1966705

**Published:** 2026-10-08

**Authors:** Eman A. Alturki, Caroline S. Weckerle, Caroline Maake, Raha Orfali, Michael Heinrich

**Affiliations:** 1 Department for Systematic and Evolutionary Botany and Botanical Garden, University of Zurich, Zurich, Switzerland; 2 Department of Pharmacognosy, College of Pharmacy, King Saud University, Riyadh, Saudi Arabia; 3 Institute of Anatomy, University of Zurich, Zurich, Switzerland; 4 Pharmacognosy and Phytotherapy, UCL School of Pharmacy, University College London, London, United Kingdom; 5 Department of Pharmaceutical Sciences and Chinese Medicine Resources, Chinese Medicine Research Center, College of Chinese Medicine, China Medical University, Taichung, Taiwan

**Keywords:** *Boswellia*, boswellic acids, dermatology, frankincense, psoriasis, skin diseases, topical therapy, wound healing

## Abstract

*Boswellia* species and the resins like frankincense derived from them have long been used in traditional medical systems for the treatment of inflammatory and skin disorders owing to their diverse bioactive constituents. This review provides a comprehensive overview of the dermatological potential of *Boswellia* species and their metabolites in inflammatory and non-inflammatory skin diseases, with particular emphasis on boswellic acids. A PRISMA-guided literature search of PubMed, Scopus, and ScienceDirect identified studies on the phytochemistry, biological activities, and dermatological applications of frankincense-producing species, supplemented by information on taxonomy and traditional uses. The genus *Boswellia* comprises more than 20 recognized species distributed across the Indian subcontinent, the Arabian Peninsula, and the Horn of Africa. Their oleo-gum resins are rich in pentacyclic triterpenes, particularly 11-keto-β-boswellic acid (KBA) and 3-*O*-acetyl-11-keto-β-boswellic acid (AKBA), which exert anti-inflammatory, antioxidant, antimicrobial, immunomodulatory, and wound-healing activities. Preclinical studies demonstrate that *Boswellia*-derived preparations attenuate key pathogenic pathways in psoriasis, eczema, and atopic dermatitis, promote wound healing, and protect against radiation and burn-induced skin injury. Emerging evidence also supports potential applications in skin aging, pigmentation disorders, and melanoma. Clinical studies suggest that topical *Boswellia* formulations may improve psoriasis, eczema, photoaging, striae distensae, and radiotherapy induced skin reactions; however, the available evidence is limited by small sample sizes, short treatment durations, heterogeneous methodologies, and variability in species, extraction procedures, and formulations. Overall, *Boswellia* species, particularly *B. sacra*, *B. serrata*, *B. papyrifera*, and *B. carterii*, represent promising multi-target candidates for dermatological applications. Future research should prioritize sustainable sourcing, phytochemical standardization, species-specific comparisons, and well-designed clinical trials to establish efficacy, optimal formulations, dosing strategies, and long-term safety.

## 1 Introduction

Skin diseases are common conditions that affect individuals across all age groups, from neonates to the elderly (Marks and Miller, 2006). Globally, skin and subcutaneous diseases constitute a substantial public-health burden, with an estimated 4.69 billion incident cases and 41.9 million disability adjusted life years (DALYs) in 2021 (World Health Organization, 2025). They are also among the top five reasons people seek medical care. In both developed and industrial nations, skin disorders contribute significantly to the healthcare burden (De Wet et al., 2013).

There are various types of skin disorders, including inflammatory skin diseases such as acne, eczema, dermatitis, and psoriasis; bacterial infections like folliculitis, cellulitis, and impetigo; viral infections such as herpes simplex, warts, chickenpox, and measles; and parasitic infections like scabies and lice. Fungal infections, including ringworm, are also common, as are tumours and cancers like melanoma and squamous cell carcinoma. Other skin conditions include burns, wrinkles, and moles (Salonki, 2011; Tabassum and Hamdani, 2014). While both synthetic and herbal medications are used to prevent and treat skin diseases, natural remedies derived from plants are widely used in the management of skin disorders and continue to attract increasing research interest (Yuan et al., 2016; Jones et al., 2021; Agrawal et al., 2024). Numerous studies have investigated the pharmacological activities and preclinical therapeutic potential of herbal products for dermatological disorders using *in vitro* and *in vivo* models (Kombate and Metowogo, 2023).

In traditional medicine, plants have long been used to manage a variety of skin conditions. Alongside commercially traded herbal remedies, many species from local floras are also employed. With chronic skin disorders such as alopecia, genital warts, vitiligo, and psoriasis often proving resistant to standard therapies, research on medicinal plants has expanded significantly in recent years (Males et al., 2019).

In recent years, interest in the traditional medicinal uses of *Boswellia* species for the treatment of skin diseases has grown considerably. A growing body of *in vitro*, *in vivo*, and clinical evidence suggests that *Boswellia* exhibits promising therapeutic potential in various skin conditions, including psoriasis, eczema, and wound healing. Despite these advances, no comprehensive review has systematically integrated the taxonomic, biological, and pharmacological aspects of *Boswellia* while highlighting the specific roles of different species and their bioactive compounds in dermatological applications. Therefore, this review aims to fill this gap by combining systematic and narrative approaches to provide a comprehensive overview of the current evidence on *Boswellia* species and their active metabolites in skin-related conditions. Furthermore, it seeks to identify current knowledge gaps and highlight key priorities for future research to support the development of *Boswellia*-based therapies for dermatological diseases.

## 2 Methodology

### 2.1 Search strategy

A literature search was conducted following the PRISMA guidelines (Figure 1) to identify biological, pharmacological and phytopharmacological studies of *Boswellia* on skin related conditions. In addition, a general electronic and hand searches focusing on the taxonomy, distribution, phytochemistry, traditional and common uses of the taxon were conducted.

**FIGURE 1:**
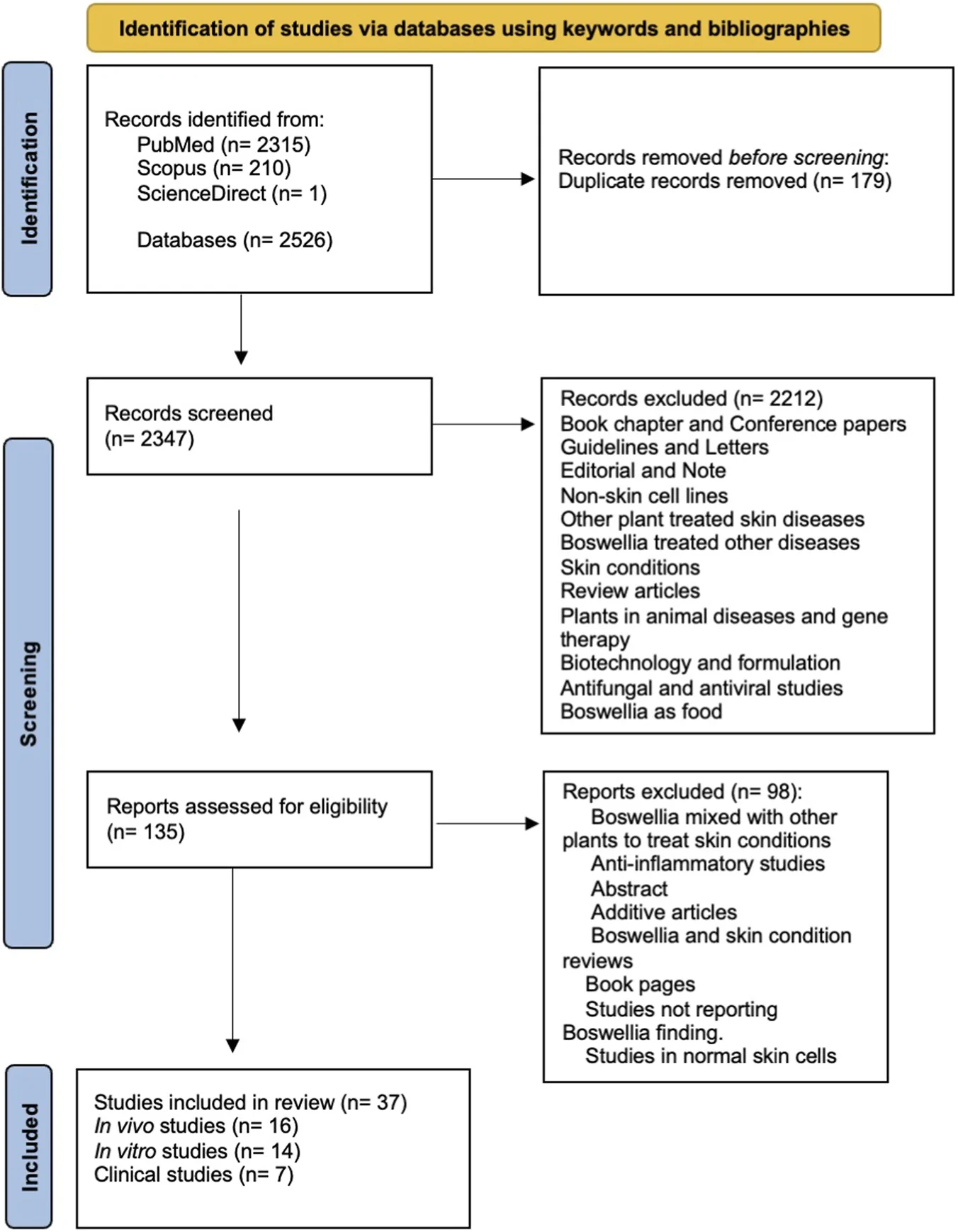
Flow diagram for inclusion and exclusion criteria in the review using PRISMA model.

To understand the role of *Boswellia* in preclinical and clinical skin conditions we performed a broad database search covering biological, pharmacological and phytopharmacological aspects. The main databases included were PubMed, Scopus and ScienceDirect covering studies up to June 2026. The search terms used were {*Boswellia* or boswellic $ or frankincense or Luban or Weihrauch} and {skin}. Publications in multiple languages, primarily English, but also German, Chinese, French, Russian, and Italian, were considered for inclusion. Where available, the English version of a publication was used. In cases where no English version was available, AI-assisted translation was employed to translate the publication into English for screening and data extraction.

In addition, following the electronic database search, an additional manual search was conducted using the above and additional keywords to ensure that all eligible studies were identified. This retrieved additional studies that met the predefined inclusion criteria and were subsequently included in the review. The database search identified a total of 2,526 records, including 2,315 records from PubMed, 210 from Scopus, and one from ScienceDirect. After removing 179 duplicate records, 2,347 records were screened based on their titles and abstracts. Of these, 2,212 records were excluded, and 135 reports were assessed for eligibility. Following full-text assessment, 98 reports were excluded for not meeting the eligibility criteria, resulting in 37 studies being included in the review. The included studies comprised 16 *in vivo* studies, 14 *in vitro* studies, and seven clinical studies. The study-selection process and reasons for exclusion are summarized in the PRISMA flow diagram (Figure 1).

### 2.2 Eligibility criteria

Studies were considered eligible for the systematic evidence synthesis if they were original studies investigating *Boswellia* species, *Boswellia*-derived preparations, frankincense, or boswellic acids in relation to skin conditions or dermatologically relevant outcomes. Eligible evidence included clinical studies and relevant *in vivo* and *in vitro* experimental studies. The presence of a comparator or control group was not required for inclusion. Publications in multiple languages, primarily English, but also German, Chinese, French, Russian, and Italian, were considered for inclusion. Where available, the English version of a publication was used. In cases where no English version was available, AI-assisted translation was employed to translate the publication into English for screening and data extraction. Grey literature was not systematically searched. Studies were excluded if they investigated *Boswellia* exclusively in non-dermatological conditions, evaluated other plants without providing specific findings for *Boswellia*, or did not report skin-related outcomes. Reviews, books and book chapters, conference papers, guidelines, letters, editorials, notes, and abstract-only publications were excluded from the systematic evidence synthesis. Studies evaluating multi-herbal preparations in which the specific contribution of *Boswellia* could not be determined were also excluded. Review articles and other secondary sources were used, where appropriate, only to provide background information on taxonomy, distribution, traditional uses, phytochemistry, and general pharmacological aspects and were not included among the 37 studies in the systematic evidence synthesis.

### 2.3 Methodological quality assessment

Given the heterogeneity of the included studies, methodological quality was assessed narratively. For clinical studies, key features including randomization, blinding, allocation concealment, sample size, and use of a comparator were considered where applicable. For animal studies, sample size, control groups and randomization, were considered. For *in vitro* studies, the use of appropriate controls, tested concentrations, exposure duration, and characterization of the tested preparation were considered. For *in silico* studies, the computational approach and validation procedures were considered. Information not provided in the original studies was recorded as “not reported.”

## 3 Results and discussions

### 3.1 Taxonomy and traditional uses of *Boswellia* spp.

The genus *Boswellia* (Burseraceae) comprises more than 20 recognized species, which are widely distributed across the Indian subcontinent, the southern Middle East, West and East Africa, and the Horn of Africa, with Socotra Island harboring nearly half of the known species (Iram et al., 2017; Al-Harrasi et al., 2018; World Flora Online, 2026; POWO, 2026). Their geographical distribution, selected traditional uses and conservation status are summarized in Table 1. The taxonomy of *Boswellia* is continuously evolving, with several species having been added or reclassified in recent years, while genetic studies continue to provide new insights into species relationships (Johnson et al., 2022).

**TABLE 1:** Distribution, conservation status of different *Boswellia* species and their traditional medicinal uses.

Species name	Distribution	Conservation status	Traditional uses	References
*B. ameero* Balf.f.	Socotra	Endangered	Bronchitis, asthma, common cold and rheumatism	POWO; Mothana and Lindequist, 2005; World Conservation Monitoring Centre (2026)
*B. asplenifolia* (Balf.f.) Thulin Synonyms: *Boswellia socotrana* subsp. *asplenifolia* (Balf.f.) Lvončık	Socotra	Endangered	Not reported	POWO; World Conservation Monitoring Centre (2026)
*B. bullata* Thulin	Socotra	Endangered	Not reported	POWO; World Conservation Monitoring Centre (2026)
*B. dalzielii* Hutch.	Nigeria, Mali, Burkina Faso, Cameroon, Benin and Togo	Least concern	Yellow fever, malaria and stomach diseases	Ouedraogo et al., 2006; World Conservation Monitoring Centre (2026)
*B. dioscoridis* Thulin,	Socotra	Endangered	Not reported	POWO; World Conservation Monitoring Centre (2026)
*B. elongata* Balf.f.,	Socotra	Endangered	Bronchitis, asthma, common cold and rheumatism	Mothana and Lindequist, 2005; World Conservation Monitoring Centre (2026)
*B. frereana* Birdw.	Somalia	Not assessed	Not reported	POWO
*B. globosa* Thulin	Somalia	Not assessed	Not reported	POWO
*B. microphylla* Chiov.	Ethiopia, Somalia, Kenya	Not assessed	Not reported	POWO
*B. nana* Hepper	Socotra	Critically Endangered	Not reported	POWO; World Conservation Monitoring Centre (2026)
*B. neglecta* S. Moore Synonyms: *Boswellia elegans* Engl. ; *Boswellia hildebrandtii* Engl. ; *Boswellia holstii* Engl. ; *Boswellia multifoliolata* Engl.	Ethiopia	Not assessed	Bronchitis, cough, cold, congestion and respiratory issues.	Fanta et al, 2013; POWO
*B. occulta* Thulin, DeCarlo & S.P. Johnson	Somalia	Not assessed	Not reported	POWO
*B. ogadensis* Vollesen	Ethiopia	Critically Endangered	Not reported	POWO; World Conservation Monitoring Centre (2026)
*B. ovalifoliolata* N.P. Balakr. & A.N.Henry	India	Vulnerable	Pain and stomach ulcer	Savithramma et al., 2011; World Conservation Monitoring Centre (2026)
*B. papyrifera* (Caill.) Hochst. Synonyms: *Boswellia chariensis* Guillaumin, *Boswellia occidentalis* Engl., *Boswellia odorata* Hutch.	Ethiopia, Sudan, and Eriteria	Not assessed	Arthritis	Ahmed et al., 2013; POWO
*B. pirottae* Chiov.	Ethiopia	Vulnerable	Not reported	POWO; World Conservation Monitoring Centre (2026)
*B. popoviana* Hepper	Socotra	Endangered	Not reported	POWO; World Conservation Monitoring Centre (2026)
*B. rivae* Engl. Synonyms: *Boswellia boranesis* Engl.; *Boswellia ruspoliana* Engl.	Ethiopia, Somalia, Kenya	Least Concern	Insect repellent	DeCarlo et al., 2023; POWO; World Conservation Monitoring Centre (2026)
*B. sacra* Flück. Synonyms: *Boswellia bhaw-dajiana* Birdw.; *Boswellia bhaw-dajiana* var*. serrulate* Engl. *Boswellia carterii* Birdw.; *Boswellia carterii* var*. subintegra* Engl.; *Boswellia carterii* var*. undulatocrenata* Engl.; *Boswellia undulatocrenata* Engl.	Arabian Peninsula (Oman and Yemen)	Near threatened	Cold symptoms, inflammatory diseases, menstrual pain, sore throat, muscle pain and arthritis	Khalifa et al., 2023; POWO; World Conservation Monitoring Centre (2026)
*B. samhaensis* Thulin & Scholte	Socotra	Critically Endangered	Not reported	POWO; World Conservation Monitoring Centre (2026)
*B. scopulorum* Thulin	Socotra	Critically Endangered	Not reported	POWO; World Conservation Monitoring Centre (2026)
*B. serrata* Roxb. Synonyms: *Boswellia glabra* Roxb.; *Boswellia hirsute* Sm.; *Boswellia serrata* var. *bivalvis* Kuntze; *Boswellia serrata* var. *glabra* (Roxb.) A. W. Benn; *Boswellia thurifera* Roxb.	India	Least Concern	Rheumatoid arthritis, Bronchial asthma, chronic inflammatory bowel diseases and psoriasis	Abdel-Tawab et al., 2011; Efferth and Oesch 2022; POWO; World Conservation Monitoring Centre (2026)
*B. socotrana* Balf.f.	Socotra	Vulnerable	Not reported	POWO; World Conservation Monitoring Centre (2026)

* Note: “Not reported” indicates that no traditional use information was identified in the sources reviewed.

*Boswellia* has long been used in traditional African, Arabian, and Indian medicine for the treatment of a wide range of diseases, primarily owing to its anti-inflammatory, antiviral, immunomodulatory, antidiabetic, and antibacterial properties (Bongers et al., 2019; Efferth and Oesch, 2022; Banno et al., 2006; al-Saidi et al., 2012; Al-Yasiry and Kiczorowska, 2016). In addition, *Boswellia* has traditionally been used to promote skin health and has gained increasing attention as a potential therapeutic agent for various dermatological conditions (Han et al., 2017).

Different *Boswellia* species exhibit diverse traditional uses depending on their geographical distribution and cultural background (Table 1). Among the most important ones are *B*. *sacra* (synonym *B. carterii*), which is distributed in the Arabian Peninsula (Oman and Yemen) and Somalia, and *B*. *serrata* originating from India. *B. papyrifera* is a widely distributed plant in Ethiopia, Eritrea, and Sudan and *B*. *frereana* is found in Northern Somalia (Elhaddad et al., 2023; POWO).

The term frankincense refers to the oleo-gum-resin obtained from several *Boswellia* species. For thousands of years, people have been collecting the resins by scraping off natural exudate or creating tiny knife cuts into the bark, waiting for the resin to seep out, allowing it to dry, scraping the “tears” and then reopening the wound to begin the harvest cycle again (Johnson et al., 2024). *Boswellia* species are used in an array of coating material, adhesives and cosmetic preparations (Roy et al., 2019).

*B*. *sacra* (often referred to by its synonym *B. carterii*) is used worldwide in various religious ceremonies, as well as in social, religious and other cultural practices (Al-Harrasi et al., 2019) and today especially due to the high use in global bioeconomies (Mykhailenko et al., 2025) is a ‘Near Threatened’ species (Table 1). It was used by the ancient Egyptians in painting, perfumes, chewing gum and beverages (Al-Saidi et al., 2012; Miran et al., 2022). *B. sacra* have been used in the Middle east traditionally for various health conditions (Al-Yahya et al., 2020). Also, in recent years, owing to its anti-inflammatory properties and its potential to promote skin health, frankincense has gained increasing attention as a medicinal product in Europe (Han et al., 2017; Mishra et al., 2020). It is used in pharmaceutical preparation, food, beverages, perfumes, cosmetics and detergents (Al-Saidi et al., 2012; El-Nagerabi et al., 2014). In Arab, African and Middle East countries *B. sacra* is used as a chewing gum because of its flavour. It is also used as a water extract of the gum resin for stomach and liver ailments and against cough (Asad and Alhumoud, 2015).

*B*. *serrata* has longstanding cultural importance because of its aromatic gum-resin. More broadly, frankincense obtained from *Boswellia* species has been used as incense in religious and cultural ceremonies since the beginning of written history and was historically valued by several ancient civilizations, including Greek and Roman societies (Moussaieff and Mechoulam, 2009).

Several African and Socotran species have primarily been associated with the treatment of respiratory conditions, inflammatory disorders, and other traditional health applications. In India, *B. serrata* is the most extensively studied species and is traditionally used for inflammatory diseases, including arthritis, asthma, inflammatory bowel diseases, and psoriasis (Sahoo et al., 2020), similarly to the use of *B. sacra* from the Arabian Peninsula. Although numerous *Boswellia* species have been documented in traditional medicine, many remain insufficiently investigated. Among the most extensively studied frankincense-producing species are *B. sacra, B. serrata,* and to a lesser degree *B. papyrifera* and *B. frereana* (Miran et al., 2022; Elhaddad et al., 2023).

### 3.2 Bioactive metabolites of *Boswellia* species

Frankincense resin is primarily composed of essential oils, triterpenes, and gum (polysaccharides) (Elhaddad et al., 2023). The essential oil fraction (approximately 5%–15% of the resin) contains a variety of mono-, di-, and triterpenes, while the resin fraction is rich in pentacyclic triterpenes, particularly boswellic acids. Boswellic acids are the most extensively studied metabolites of *Boswellia* species and are considered the principal bioactive compounds responsible for many of the pharmacological effects attributed to frankincense (Ammon, 2006; Mertens et al., 2009; Sharma et al., 2009). For their structures see Figure 2. Although both tetracyclic and pentacyclic triterpenes are present in *Boswellia* resins, the content of boswellic acids varies considerably between species: *B. frereana* contains only trace amounts, whereas *B. papyrifera, B. serrata*, and *B. sacra* are characterized by substantially higher levels (Elhaddad et al., 2023; POWO). An important source of heterogeneity across the available studies is the use of different *Boswellia*-derived preparations, including resin extracts, essential oils, fixed oils, isolated boswellic acids, synthetic derivatives, and formulated topical products. These preparations should not be considered pharmacologically interchangeable because their chemical composition and concentrations of bioactive constituents may differ considerably. In particular, the content of individual boswellic acids, including KBA and AKBA, was not consistently reported across studies. Such variability may influence the observed pharmacological activity and complicates direct comparison and clinical interpretation of the findings. Therefore, detailed phytochemical characterization and standardization of *Boswellia*-derived preparations are important for future preclinical and clinical studies.

**FIGURE 2:**
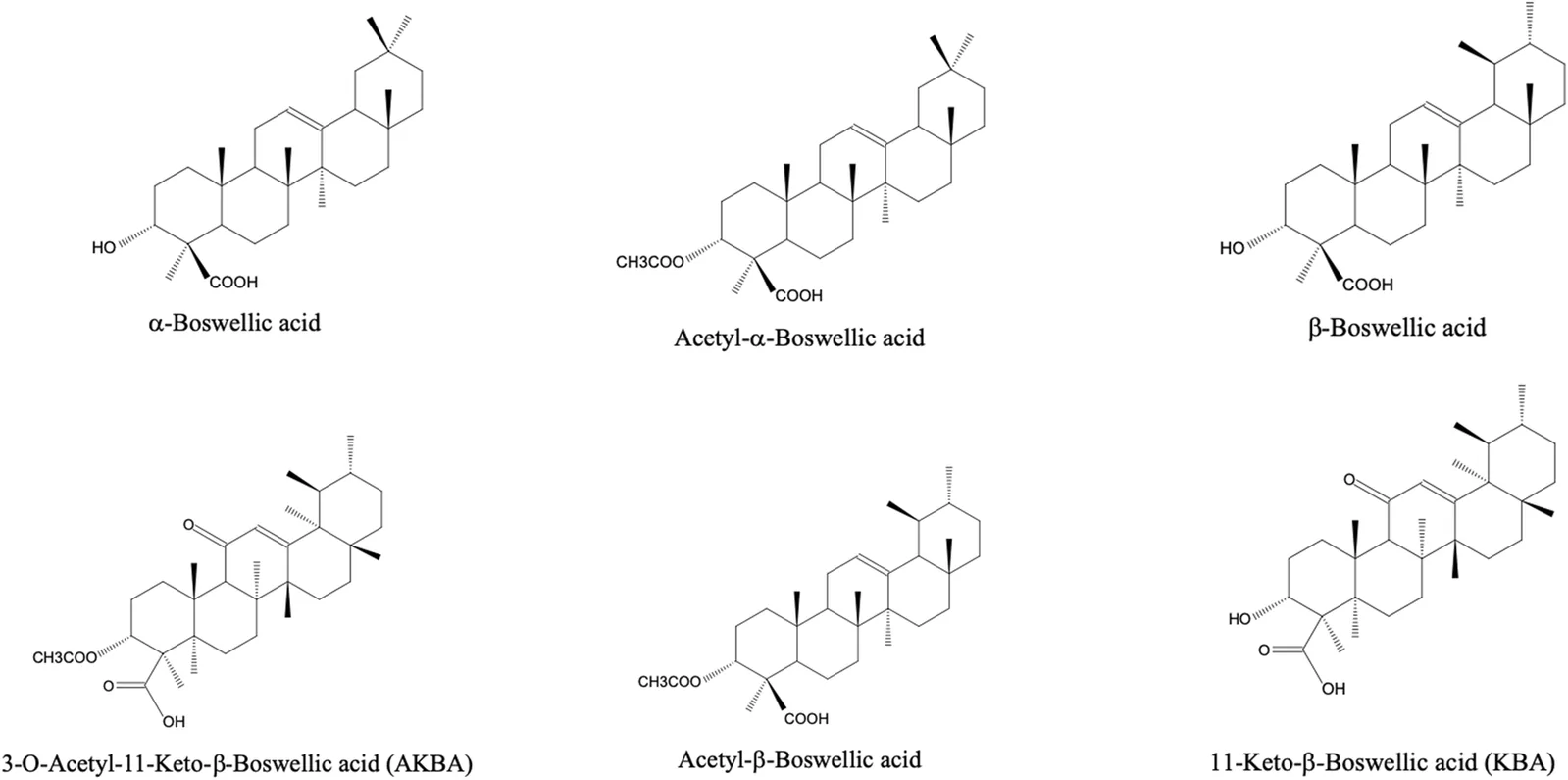
Chemical structures of main boswellic acids.

### 3.3 Pharmacological activities of *Boswellia* species and boswellic acids and their therapeutic potential in dermatology

#### 3.3.1 Pharmacological activities of boswellic acids

##### 3.3.1.1 Anti-inflammatory activity

Boswellic acids are the principal bioactive metabolites of *Boswellia* and are widely recognized for their anti-inflammatory properties. The major boswellic acids include α- and β-boswellic acid (α-BA and β-BA), acetyl-α- and acetyl-β-boswellic acid (AαBA and AβBA), KBA and AKBA (Figure 2) (Shah et al., 2009; Togni et al., 2014; Ammon, 2019). All are pentacyclic triterpene acids (Wu et al., 2024). Among them, KBA and particularly AKBA exhibit the strongest anti-inflammatory activity (Siddiqui, 2011; Alluri et al., 2019).

The concentration of individual boswellic acids varies considerably among *Boswellia* species and according to their geographic origin. For example, African *Boswellia* samples generally contain lower levels of KBA than AKBA, whereas Indian samples contain approximately equal amounts of both compounds (Ammon, 2006).

AKBA is considered the most pharmacologically active boswellic acid because of its potent inhibitory effects on inflammatory cells and mediators, resulting in reduced inflammatory activity. Numerous studies have elucidated the molecular mechanisms underlying boswellic acids’ anti-inflammatory effects. These compounds directly inhibit key inflammatory targets, including 5-lipoxygenase (5-LOX) and microsomal prostaglandin E synthase-1 (mPGES-1), thereby suppressing the biosynthesis of pro-inflammatory mediators (Cui et al., 2024; Börner et al., 2021). In particular, inhibition of 5-LOX prevents the conversion of arachidonic acid into pro-inflammatory leukotrienes. Boswellic acids exert anti-inflammatory effects through mechanisms that differ from those of non-steroidal anti-inflammatory drugs (NSAIDs), including modulation of 5-lipoxygenase and other inflammatory pathways (Abdel-Tawab et al., 2011; Cui et al., 2024; Börner et al., 2021). However, differences in mechanism of action should not be interpreted as evidence of superior safety, particularly because direct comparative clinical data between *Boswellia*-derived topical preparations and systemic NSAID therapy are lacking.

##### 3.3.1.2 Antimicrobial activity

AKBA also exhibits the strongest antibacterial activity, particularly against Gram-positive bacteria, with minimum inhibitory concentrations (MICs) ranging from 2 to 8 µg/mL (Raja et al., 2011a). Against *Staphylococcus aureus* ATCC 29213, AKBA demonstrated bacteriostatic activity, producing an approximately 2-log10 reduction in bacterial viability at 4× and 8× the MIC over 24 h (Raja et al., 2011a). Mechanistic studies indicate that AKBA disrupts bacterial membrane integrity (Raja et al., 2011a). Treatment with AKBA increased propidium iodide uptake by *S. aureus* cells, suggesting enhanced membrane permeability due to structural damage (Raja et al., 2011a). In addition, intracellular components absorbing at 260 and 280 nm, supporting disruption of the bacterial membrane permeability barrier (Raja et al., 2011a). AKBA has also been shown to inhibit biofilm formation by several Gram-positive oral and opportunistic pathogens, including *Actinomyces viscosus*, *S. mutans*, *S. aureus*, and *S. epidermidis*, and to reduce the biomass of established biofilms (Raja et al., 2011a; Raja et al., 2011b).

##### 3.3.1.3 Cytotoxic and anticancer activity

Boswellic acids have demonstrated cytotoxic and anticancer-related effects in experimental cancer models. In highly metastatic B16F10 melanoma cells, boswellic acid acetate (BC-4) inhibited proliferation, induced marked melanocytic differentiation, reduced cell migration, induced G1-phase cell-cycle arrest, and decreased topoisomerase II activity (Zhao et al., 2003). Additionally, 3-O-acetyl-*β*-boswellic acid (*β*-ABA) has been reported to exert selective cytotoxic effects, showing lower toxicity toward normal cells while inducing DNA fragmentation in melanoma cells (Kumar et al., 2016). Boswellic acids, particularly AKBA, have demonstrated antitumor effects in various cancer cell models, including colon, prostate, leukocyte, liver, and brain tumor cells (Shen et al., 2012). Depending on the cancer type and cellular context, boswellic acids may inhibit tumor progression and metastasis through multiple mechanisms, including suppression of angiogenesis, induction of apoptosis, downregulation of AKT phosphorylation, and inhibition of tumor vascularization (Liu et al., 2002; Uthaman et al., 2012). However, these findings are predominantly preclinical and do not establish clinical anticancer efficacy of boswellic acids in melanoma or other cancers.

#### 3.3.2 Dermatological applications of *Boswellia* species and boswellic acids

##### 3.3.2.1 Anti-inflammatory skin disorders

*Boswellia* species and their bioactive compounds have been most extensively investigated for inflammatory skin disorders, reflecting their well-established anti-inflammatory properties (Table 2 and 3). Boswellic acids, particularly AKBA and related derivatives, have been shown to modulate several inflammatory pathways, including 5-lipoxygenase activity, NF-κB signaling, MAPK activation, and the production of pro-inflammatory cytokines. These mechanisms suggest a potential therapeutic role of *Boswellia*-derived compounds in chronic inflammatory dermatoses.

**TABLE 2:** *Boswellia* species and boswellic acids investigated in eczema and psoriasis.

Species/Compound	Preparation	Type of study	Dose/Duration	Outcome/Effect	References
*B. serrata*	Bosexil® *Boswellia serrata* extract, water, glycerin, polyacrylamide, C13–14 isoparaffin, and laureth-7, hydrogenated polydecene, caprylic/ capric triglyceride, lecithin, tocopherol, ascorbyl palmitate, citric acid and phenoxyethanol	39 patients in randomized, placebo-controlled, double-blind trial (psoriasis or erythematous eczema)	Topical, twice daily for 30 days [dose not reported]	Marked improvements in psoriasis and erythematous eczema were demonstrated using PASI and EASI scores together with clinical grading, with psoriasis scales improving in 70% of patients and erythema in 60%, while in erythematous eczema, both itch and erythema improved in 60% of patients.	Togni et al., 2014
*B. serrata*	Isopropyl myristate in water and 36% Tween 80, 9% ethanol and 1% Carbopol 934	*In vivo*, Imiquimod-induced psoriasis-like mouse model; n = 24	Topical, 2% w/w *B. serrata* hydro-alcoholic extract in a 10 days treatment [dose not reported]	Reduction in PASI-like scores for erythema, thickness (infiltration) and scaling, with overall improvement of psoriasis-like ear lesions	Fereidouni et al., 2025
3-O-cyclohexanecarbonyl-11-keto-*β*-boswellic acid	5% lightweight liquid paraffin, 5% isopropyl myristate, 0.02% vitamin E and 89.98, 89.9, 89.5% and 89% of white vaseline	*In vivo*, Imiquimod-induced psoriasis-like mouse model; n = 48	Topical, 0.02, 0.1, 0.5% and 1% CKBA once daily for 10 days [dose not reported]	Improvement in clinical scores for scaling, erythema, and inflammatory skin changes	Lou et al., 2024
Frankincense [unspecified]	Diterpenoid derivative and boswellic acids	*In silico*	Diterpenoids and triterpenoids	Antipsoriatic effect through psoriasis-related proteins	Halim et al., 2020
Acetyl-11-keto-*β-*boswellic acid	Dissolved in DMSO and diluted with water (final conc. < 0.1% DMSO orally)	*In vivo*, Imiquimod-induced psoriasis-like mouse model; n = 48	Oral, 25,50 and 100 mg/kg/day for 6 days	Lower PASI score, indicating improvement in psoriasiform skin lesions	Wang et al., 2018
Acetyl-11-keto-*β-*boswellic acid	AKBA stock was dissolved in DMSO, then diluted into RPMI-1640 culture medium containing 10% fetal bovine serum	*In vitro*, murine bone marrow–derived dendritic cells	0.625,1.25 and 2.5 μg/mL up to 24 h	Reduced IL-12/IL-23 production, and limited their ability to drive CD4^+^ T-cell proliferation and differentiation, likely *via* inhibition of TLR8–MyD88–IRF3 signalling, supporting an anti-psoriatic immunomodulatory effect	Wang et al., 2018
*B. carterii*	Frankincense oil extract [unspecified]	*In vivo*, imiquimod-induced murine psoriasis-like dermatitis mouse model; n = 35	Topical, daily for 8 days [dose not reported]	Improved PASI scores** **and skin appearance	Li et al., 2026

**TABLE 3:** *Boswellia* species and boswellic acids investigated in atopic dermatitis.

Species/Compound	Preparation	Type of study	Dose/Duration	Outcome/Effect	References
*α*-boswellic acid	Sigma-Aldrich (St. Louis, Mo, USA) [unspecified]	*In vivo*, atopic dermatitis mouse model [number of animals not reported]	Topical, 3 and 10 mg/kg 1-2 times daily for multiple consecutive days	Combined improvement of skin barrier function and inflammation clinical score	Tsai et al., 2022
*α*-boswellic acid	Sigma-Aldrich (St. Louis, Mo, USA) in DMSO	*In vitro*, human keratinocyte cell line	1–50 μM for 1 h	Anti-inflammatory effect in keratinocytes relevant to atopic dermatitis	Tsai et al., 2022
3-O-cyclohexanecarbonyl-11-keto-*β*-boswellic acid (synthesized in the lab from AKBA)	1% *w/w* CKBA [unspecified]	*In vivo*, atopic dermatitis mouse model; n = 10	Topical, 25 mg once daily for 10 days	Reduce ear thickness, skin barrier dysfunction, epidermal hyperplasia, collagen loss and IgE serum level.	Tong et al., 2023
3-O-cyclohexanecarbonyl-11-keto-*β*-boswellic acid (synthesized in the lab from AKBA)	5 μm in DMSO vehicle [unspecified]	*In vitro*, bone marrow–derived mast cells	5 μm [duration not reported]	Inhibited IgE/FcεRI-mediated mast cell degranulation and ERK-dependent production of histamine, PGD_2_, and LTC_4_	Tong et al., 2023
*B. sacra* volatile and fixed oil	5 mg of *B. sacra* in 1 mL of viscous topical solution	*In vivo*, acute allergic contact dermatitis rat model; n = 25	Topical, thin topical film once and assessed 3 h post treatment	Soothing, alleviating and partially healing effect through clinical scoring	Ibrahim et al., 2025

###### 3.3.2.1.1 Psoriasis and inflammatory eczema

Psoriasis and eczema are among the most systematically investigated dermatological applications of *Boswellia* (Table 2). A randomized, placebo-controlled clinical trial demonstrated that topical application of a 0.5% *B. serrata* preparation (Bosexil®) improved psoriasis-associated scaling and erythema, as well as itching and erythema in patients with erythematous eczema (Togni et al., 2014). However, findings obtained with specific commercial Bosexil® formulations may not be directly generalizable to other *Boswellia* extracts or topical formulations with different compositions and degrees of standardization. Furthermore, preclinical studies have demonstrated that boswellic acid derivatives, including AKBA and cyclohexanecarbonyl-11-keto-β-boswellic acid (CKBA), reduce psoriasis-like inflammation in animal models by suppressing IL-17A-driven inflammatory responses, inhibiting dendritic cell activation, and reducing cytokine production (Wang et al., 2018; Lou et al., 2024). Recent formulation approaches, such as *B. serrata* nanoemulsion gels, further indicate improved topical efficacy in reducing erythema, scaling, and skin thickening (Fereidouni et al., 2025). Collectively, these findings support *Boswellia*-derived topical preparations as promising multi-target agents that ameliorate key psoriatic and eczematous features.

However, the current evidence base is constrained by short treatment durations, and a lack of adequately powered trials, leaving optimal dosing, long-term safety and the translational relevance of these mechanistic pathways in diverse psoriasis and eczema phenotypes as important unresolved issues.

###### 3.3.2.1.2 Atopic dermatitis

The potential of boswellic compounds in atopic dermatitis has been investigated mainly through *in vitro* and *in vivo* models (Table 3). α-BA was shown to improve skin barrier dysfunction, reduce inflammatory cell infiltration, and suppress NF-κB and MAPK-related pathways in keratinocyte and mouse models of atopic dermatitis (Tsai et al., 2022). Similarly, CKBA topical treatment reduced epidermal hyperplasia, collagen degradation, IgE levels, and mast cell-associated inflammatory mediators in an experimental model of atopic dermatitis (Tong et al., 2023). Additionally, *B. sacra* volatile and fixed oils demonstrated protective effects against allergic dermatitis by modulating inflammatory biomarkers such as ICAM-1 and leukotriene B4 (Ibrahim et al., 2025).

However, the current evidence remains limited by the absence of controlled clinical trials, heterogeneous experimental designs and formulations, and a lack of long-term safety, leaving optimal dosing, vehicle selection and the clinical relevance of these mechanistic effects as important unresolved questions.

Overall, current evidence indicates that boswellic acids may represent promising candidates for the management of inflammatory skin diseases. However, most studies remain limited to experimental models, and further clinical investigations are required to establish optimal formulations, doses, and long-term efficacy.

##### 3.3.2.2 Skin aging, photoaging, and extracellular matrix remodeling

Beyond inflammatory disorders, *Boswellia*-derived compounds have attracted interest for their potential role in skin aging due to their effects on oxidative stress, collagen metabolism, and extracellular matrix remodeling (Table 4).

**TABLE 4:** *Boswellia* species in photoaged skin.

Species/Compound	Preparation	Type of study	Dose/ Duration	Outcome/Effect	References
*B. serrata*	0.5% boswellic acid cream [unspecified]	Randomized, double-blind, placebo-controlled split-face clinical study, 15 female participants	Topical, applied once daily for 30 days [dose not reported]	Improvement in the Dover’s global score for photoaging, primarily through reduction of fine lines and tactile roughness.	Calzavara-Pinton et al., 2010
*B. papyrifera*	0.5% of frankincense essential oil +1% Compritol® + 4% Tween®	*In vivo*, UVB-irradiated rat skin model of photodamage/photoaging [number of animals not reported]	Topical formulation, once daily for 10 days [dose not reported]	Enhanced cutaneous antioxidant defenses, modulated MAPK/TGF-β signaling, and normalized MMP/procollagen I expression, collectively indicating anti-photoaging potential.	Kotb et al., 2023
*B. papyrifera*	Hydro-distilled and microwave-assisted hydro-distilled frankincense essential oil [unspecified]	*In vitro*, anti-collagenase and anti-elastase enzyme inhibition assays	7.81–1000 μg/mL [duration not reported]	Exhibit potent anti-collagenase and anti-elastase skin activities	Kotb et al., 2023 72

A randomized split-face clinical study showed that topical application of 0.5% boswellic acid cream derived from *B. serrata* improved photoaging-related parameters, particularly fine surface lines and tactile roughness, without significant adverse effects (Calzavara-Pinton et al., 2010). These effects were attributed to the anti-inflammatory properties of boswellic acids and their potential influence on collagen and elastin metabolism.

These promising anti-aging results are limited to small, short-term studies using a single low-dose cream, so the durability of benefit, best dose and effects on deeper photoaging features, and long-term safety of *Boswellia*-based products all remain uncertain.

In addition, *B. papyrifera* essential oil demonstrated anti-aging potential through inhibition of collagenase and elastase activity, with limonene and α-pinene contributing to synergistic effects (Kotb et al., 2023). The same study showed that frankincense oil-loaded solid lipid nanoparticles provided enhanced protection against UVB-induced skin damage compared with free oil formulations.

Studies using human dermal fibroblasts further support these findings (Tables 5 and 6). Enriched *B. serrata* extracts standardized in boswellic acids reduced inflammatory markers such as TNF-α and nitric oxide, suggesting potential applications in maintaining dermal homeostasis and supporting collagen formation (Sudhakar et al., 2021). Similarly, frankincense essential oil reduced inflammation-associated markers, including ICAM-1 and IP-10, in pre-inflammatory dermal fibroblast models (Han et al., 2017).

**TABLE 5:** *Boswellia* species investigated in dermal fibroblast.

Species/Compound	Preparation	Type of study	Dose/Duration	Outcome/Effect	References
*B. serrata*	65% and 40% Ethanol extract (10 mg of extract in DMSO)	*In vitro*, human dermal fibroblast cells	100 and 200 μg/mL for 24 h	Markedly attenuates LPS-induced TNF-α and nitric oxide, outperforming regular boswellic extract, and thus shows promise as an anti-inflammatory active for skin applications	Sudhakar et al., 2021
Frankincense essential oil	FERO in DMSO	*In vitro*, primary human neonatal dermal fibroblasts	0.00011%, 0.00033%, 0.001% and 0.003% *v/v* for 24 and 72 h	Anti-proliferative activity in pre-inflamed human dermal fibroblasts and modulated biomarkers pathways related to inflammation and tissue remodelling.	Han et al., 2017

**TABLE 6:** *Boswellia* species investigated in striae distensae (*dermal scarring resulting from the stretching of the dermis)*.

Species/Compound	Preparation	Type of study	Dose/Duration	Outcome/Effect	References
*B. serrata*	Commercial cream (Pharmaland SA): *Boswellia serrata*, *Centella asiatica*, Soia phospholipids, Polyunsaturated fatty acids	Open-label, multicentre clinical study, 102 female, 2 male and 14 sexes not reported	Topical, twice daily application for 45 and 90 days [dose not reported]	Reduction in the global severity score of striae distensae over 90 days	Sparavigna et al., 2005

These fibroblast studies indicate that *Boswellia* extracts and frankincense oils can directly suppress inflammatory mediators, but their relevance to skin disease remains uncertain because they rely on simplified cell models, variable preparations and lack confirmatory human *in vivo* data.

##### 3.3.2.3 Wound healing and radiation-induced skin injury

The anti-inflammatory and tissue-protective properties of *Boswellia* may also contribute to applications in wound healing and treatment of therapy-related skin damage (Table 7 and 8).

**TABLE 7:** *Boswellia* species and boswellic acids investigated in wound healing.

Species/Compound	Preparation	Type of study	Dose/Duration	Outcome/Effect	References
*B. carterii*	70% alcoholic extract powder (5, 10, 20, and 30 g) mixed with up to 100 g vaseline	*In vivo*, Infected linear excision wound albino rabbits’ model; n = 24	Topical, daily for 23 days [dose not reported]	Markedly accelerated infected wound healing with the 5% BC within 6 days, as assessed by the wound contraction assay	Hasson et al., 2022
*B. serrata*	Extraction in 99.5 or 70% ethanol incorporated at 5% in addition to 80% sunflower oil and 15% glycerol dibehenate	*In vivo*, rat scald burn model; n = 30	Topical, 0.2 g applied once daily for 21 days	Accelerated wound closure, reduced inflammation, improved hydration, and lowered melanin (post-inflammatory pigmentation) as measured by wound surface area planimetry, TEWL, Corneometer hydration, Mexameter erythema/melanin indices, and histological scores for inflammation and epithelialization	Roşca et al., 2025
*B. serrata*	Bosexil®, 1% *Boswellia serrata* extract (*Boswellia* Phytosome®) formulated as a topical emulsion	Single blind clinical study, 30 healthy volunteers	Topical, twice daily for 10 days [dose not reported]	Faster recovery of the skin barrier, with lower Transepidermal water loss	Rigano et al., 2015
Boswellic acid	5, 10 and 20 g of boswellic acid mixed with white petroleum jelly to prepare 100 g of ointment	*In vivo*, Excisional wound model, albino rats; n = 45	Topical, once daily for 15 days [dose not reported]	Accelerated wound healing in a concentration-dependent manner, as demonstrated by both macroscopic and microscopic assessments	Marouf et al., 2025
*β*-boswellic acid	(CAS: 631-69-6, purity ≥99.89%) from MedChemExpress (MCE, No. HY-N2513), derived from *B. serrata*	*In vivo*, diabetic wound model rats [number of animals not reported]	Subcutaneous, 10 mg/kg for 21-day study period	Accelerated diabetic wound healing by enhancing wound closure, reducing inflammation, promoting collagen deposition, and inhibiting ferroptosis, as confirmed by macroscopic evaluation and histopathological analysis	Han et al., 2025
*β*-boswellic acid	(CAS: 631-69-6, purity ≥99.89%) was sourced from MedChemExpress (MCE, No. HY-N2513), derived from *B. serrata*	*In vitro*, human skin fibroblasts cells	40 μM concentration for 24 h	Improved fibroblast viability and migration while inhibiting high glucose-induced ferroptosis and oxidative stress in human skin fibroblasts.	Han et al., 2025
*α*-boswellic acid	From Nakeli Biotechnology (Chengdu, China; CAS Number: 471-66-9), dissolved in 10% DMSO and diluted with saline to a 3% solution.	*In vivo*, full-thickness excisional acute wound rats’ model; n = 24	Topical, 3% once daily application solution for 14 days	Accelerated acute wound healing, as demonstrated by macroscopic wound healing assessment and histopathological (H&E) analysis	Dong et al., 2024
*α*-boswellic acid	100 μg/ mL of *α*-boswellic acid [unspecified]	*In vitro*, human hypertrophic scar fibroblasts	100 µg/mL *α*-boswellic acid, exploratory concentrations were 100, 200, 300, and 400 µg/mL for 48 h duration	Inhibited the proliferation and migration of human hypertrophic scar fibroblasts	Dong et al., 2024
*B. carterii*	40% *B. carterii* oleo gum resin in a base of oily duck fat	Randomized double blind clinical trial for 2nd degree burn wounds, 32 patients (15 female and 17 male)	Topical, wound dressing changed every 2 days for up to 20 days [dose not reported]	Burn wound healing was comparable to 1% silver sulfadiazine, with similar healing time and wound healing index	Badr et al., 2023

**TABLE 8:** *Boswellia* species investigated in radiotherapy skin damage.

Species/Compound	Preparation	Type of study	Dose/Duration	Outcome/Effect	Reference
*B. serrata*	Bosexil®, 2% boswellic acids cream in Phytosome® formulation	Randomized, Parallel, placebo-controlled clinical trial trial in breast cancer patients undergoing adjuvant radiotherapy, 114 female participants	Topical, twice daily from the first day of radiotherapy (app. 5 weeks) [dose not reported]	Reduced radiation-induced erythema with a trend toward alleviating superficial symptoms (itching and burning), as assessed by visual erythema grading, computer-assisted skin colour analysis, and RTOG toxicity scoring.	Togni et al., 2015

A randomized placebo-controlled clinical trial evaluated a 2% *B. serrata* cream formulation (Bosexil®) in breast cancer patients undergoing radiotherapy (Table 8) (Togni et al., 2015). Topical treatment reduced radiation-induced erythema and decreased superficial symptoms, including itching and burning sensations, suggesting a protective effect against radiation-associated skin reactions (Togni et al., 2015).

Additionally, a case report described the successful use of orally and topically administered *B. sacra* in a patient with recurrent pilonidal sinus disease (Table 9) (Al Hashmi and Harvey, 2023). Treatment resulted in wound closure, cessation of bleeding, and absence of recurrence during follow-up (Al Hashmi and Harvey, 2023). Although promising, these findings require confirmation in controlled clinical studies.

**TABLE 9:** *Boswellia* species investigated in pilonidal sinus disease.

Species/Compound	Preparation	Type of study	Dose/Duration	Outcome/Effect	Reference
*B. sacra*	3,000 mg of *B. sacra* in 50 mL water	Pilonidal sinus disease (case report; one female patient)	Oral and topical, once daily for 5 days	Complete ulcer healing and cessation of bleeding within 5 days, assessed by clinical observation and serial photographs	Al Hashmi and Harvey, 2023

##### 3.3.2.4 Antimicrobial and protective effects against skin pathogens

In studies of skin-associated microorganisms (Table 10), methanolic extracts of *B. serrata* demonstrated antibacterial activity against *S. aureus* and *Listeria monocytogenes* with stronger effects observed against Gram-positive bacteria (Todorović et al., 2025). Similarly, *B. sacra* extracts exhibited antibacterial activity against *S. aureus*, *B. cereus*, *P. aeruginosa*, and *E. coli* (Al-Saban et al., 2025). These studies primarily evaluated activity against planktonic microorganisms; biofilm formation or established biofilms were not evaluated.

**TABLE 10:** *Boswellia* species investigated in skin pathogens.

Species/Compound	Preparation	Type of study	Dose/Duration	Outcome/Effect	Reference
*B. serrata*	1 g resin extracted with 10 mL methanol	*In vitro*, *S*. *aureus*, *L*. *monocytogenes*, and *P. aeruginosa*	10 mg/mL (1.5 h incubation)	Antibacterial activity, particularly against *S. aureus* and *L. monocytogenes*, with weaker activity against *P. aeruginosa*	Todorović et al., 2025
*B. sacra*	Ethanolic resin extract prepared by maceration (200 g resin in 300 mL 96% ethanol for 72 h) and** **Soxhlet extraction (50 g resin in 300 mL 96% ethanol at ∼80 °C for 6 h); extract formulated into a topical cream containing 2 g Soxhlet extract	*In vitro*, *S. aureus, B. cereus, P. aeruginosa,* and *E. coli, Candida albicans*	100, 75, 50 and 25 mg/mL, incubated 24 h** **for bacteria (48 h for *Candida albicans*)	Dose-dependent antibacterial activity, particularly against *S*. *aureus*, *B*. *cereus*, *P. aeruginosa*, and *E*. *coli*.	Al-Saban et al., 2025

These findings provide preliminary evidence of the antibacterial activity of *Boswellia*-derived preparations against several skin-associated microorganisms. However, the available evidence is predominantly based on *in vitro* studies, and direct comparisons with standard topical antimicrobials are lacking. Therefore, the clinical usefulness of these findings for the prevention or treatment of dermatological infections cannot yet be established. Further studies are required to determine clinically relevant concentrations, appropriate topical formulations, and skin safety profiles.

##### 3.3.2.5 Emerging applications: pigmentation disorders and melanoma

Recent research has expanded the potential dermatological applications of *Boswellia* beyond inflammatory and regenerative processes (Table 11). Frankincense volatile oil demonstrated antioxidant, anti-inflammatory, and pigmentation-modulating effects in zebrafish models by reducing melanin production, reactive oxygen species, and neutrophil aggregation (Yan et al., 2024). However, these findings are exploratory and are limited to zebrafish and *in vitro* models. The authors highlight that formulation challenges (poor solubility and dark colour), lack of acute and chronic toxicity data, and the need for multi-omics validation and clinical studies remain unresolved before frankincense volatile oil can be translated into a potential pigmentation-modulating agent (Yan et al., 2024), which however, is at a very early stage and several of the methods used are of no or very limited pharmacological relevance.

**TABLE 11:** Frankincense volatile oil investigated in pigmentation disorders.

Species/Compound	Preparation	Type of study	Dose/ Duration	Outcome/Effect	References
Frankincense volatile oil	0.2 g in 99.8 butylene glycol	*In vivo*, zebrafish embryos, n = 12/group	200 μL of 5%, 50% and 100% for 48 h	Inhibition of melanin synthesis in zebrafish embryos, as assessed by melanin inhibition test (immersion in culture medium)	Yan et al., 2024
Frankincense volatile oil	0.2 g in 99.8 butylene glycol	*In vivo*, zebrafish embryos; n = 24	5%, 50% and 100% (40 min exposure)	Reduced neutrophil aggregation, as assessed by anti-inflammatory assay	Yan et al., 2024
Frankincense volatile oil	0.2 g in 99.8 butylene glycol	*In vivo*, zebrafish embryos; n = 24	5% (24 h exposure)	Reduced intracellular reactive oxygen species (ROS), as assessed by ROS elimination assay	Yan et al., 2024
Frankincense volatile oil	FVO in Ethanol	*In vitro*, cell-free DPPH assay	500–5,000 μg/mL, 30 min incubation	Dose-dependent antioxidant activity, as assessed by the DPPH radical scavenging assay	Yan et al., 2024

Furthermore, boswellic acid derivatives have demonstrated preliminary anticancer related activity in melanoma models (Table 12). Boswellic acid acetate and 3-O-acetyl-β-boswellic acid inhibit melanoma cell migration, induce apoptosis, and affect cell-cycle progression *in vitro* (Zhao et al., 2003; Jamshidi-Adegani et al., 2023). Although these findings are preliminary and do not establish clinical anticancer efficacy in melanoma, Further preclinical and clinical investigations are required to determine their therapeutic relevance.

**TABLE 12:** Boswellic acids investigated in melanoma.

Species/Compound	Preparation	Type of study	Dose/ Duration	Outcome/Effect	References
BC-4 (isomer compound isolated from *B. carterii* containing 1:1 *α* and *β*- boswellic acid acetate)	10 mg in 1 mL ethanol	*In vitro*, mouse melanoma cells	12.5. and 25 μM for 48 h	Reduced melanoma cell migration and metastatic potential by inducing G1 cell-cycle arrest, promoting differentiation, inhibiting topoisomerase II activity, and suppressing MMP secretion	Zhao et al., 2003
3-O-acetyl-*β*-boswellic acid	*β*-ABA-loaded 3D-printed polycaprolactone (PCL) scaffold for sustained local drug delivery	*In vitro*, melanoma and fibroblast cells	Sustained release for 14 days	Selective anti-melanoma activity with minimal toxicity to fibroblasts; induced apoptosis by upregulating P53, downregulating BCL2, and increasing the Bax/BCL2 ratio	Jamshidi-adegani et al., 2023

#### 3.3.3 Research gaps and future perspectives

Although increasing evidence supports the dermatological potential of *Boswellia* species, current studies remain limited by small sample sizes, short treatment durations, and a predominance of *in vitro* and animal models. The methodological quality and reporting of the included studies were also heterogeneous, particularly regarding sample size, randomization, blinding, controls, and reporting of experimental conditions. These limitations should be considered when interpreting the overall robustness of the available evidence. Boswellic acids are highly lipophilic compounds with limited aqueous solubility and are known to exhibit poor oral bioavailability. In topical dermatological applications, their local availability may be influenced by drug release from the formulation, percutaneous absorption, and the composition of the topical vehicle. Therefore, optimization of the vehicle is an important consideration for improving the dermal delivery of boswellic acids. An additional translational limitation is that concentrations producing biological effects in *in vitro* models may not necessarily be achieved within human skin following topical application. Data on dermal absorption, distribution and retention across different skin layers, and local metabolism of boswellic acids remain limited. Consequently, direct translation of experimentally effective concentrations to clinically relevant topical doses should be approached cautiously. Advanced delivery systems, including nanoemulsions, phytosome-based formulations, and lipid-based delivery systems, may improve dermal delivery and local availability of boswellic acids. However, evidence regarding their comparative skin penetration, long-term safety, formulation stability, and scalability remains limited. Safety data for topical *Boswellia*-derived preparations also remain limited and inconsistently reported across studies. Although some clinical studies have reported topical formulations to be generally well tolerated, systematic evidence regarding local irritancy, sensitization, allergic contact dermatitis, and phototoxicity remains limited. Data on cytotoxicity toward normal keratinocytes and dermal fibroblasts, systemic absorption following topical application, and potential interactions with conventional dermatological treatments are also limited. These safety considerations are particularly relevant for essential oils and concentrated resin preparations, especially when applied to inflamed, damaged, or irradiated skin. Further studies should therefore include systematic assessment and reporting of local and systemic adverse effects, skin irritation and sensitization, phototoxicity, cellular safety, systemic exposure, and concomitant treatment use. Future studies should integrate skin-permeation and pharmacokinetic assessments with pharmacological outcomes to determine whether experimentally active concentrations can be achieved safely in human skin. In addition, future research should focus on well-designed clinical trials, standardized extracts, species-specific comparisons, and improved delivery systems to determine the therapeutic value of *Boswellia*-derived compounds in dermatology.

## 4 Conclusion

This review highlights the growing evidence supporting the dermatological potential of *Boswellia* species and their bioactive metabolites, particularly boswellic acids. A core basis for any further use and development is ascertaining the sustainable sourcing especially of the resin from *B. sacra*, which is threatened due to its bioeconomical overuse, risks linked to climate change, a very low reproductive success and general low population levels (Mykhailenko et al., 2025). Current findings indicate that *Boswellia*-derived preparations exert multiple biological effects relevant to skin health, including anti-inflammatory, immunomodulatory, antioxidant, antimicrobial, and tissue-remodeling effects.

Among the dermatological indications investigated, psoriasis has received considerable preclinical attention and limited clinical evaluation. Experimental studies demonstrated that boswellic acids, particularly AKBA and CKBA, modulate psoriasis-associated inflammatory pathways, including IL-17-mediated signaling, NF-κB activation, and cytokine production. Moreover, preliminary clinical evidence with topical *B. serrata* formulations suggests improvements in psoriasis severity parameters, supporting further investigation of frankincense-based topical therapies.

Despite these promising findings, several methodological limitations are consistently observed across the available literature. Current evidence remains limited by heterogeneous study designs, small clinical cohorts, short intervention periods, and variations in *Boswellia* species, extraction methods, and concentrations. Importantly, many studies provide insufficient methodological detail regarding therapeutic plant preparations, including the exact *Boswellia* species used, the quantity of plant material, dosing, and formulation protocols, which reduces transparency and hinders reproducibility and clinical translation. Therefore, direct comparison between studies remains challenging. The lack of standardized preparations also complicates the interpretation of pharmacological effects, clinical efficacy and the identification of the most therapeutically relevant *Boswellia* species and bioactive metabolites.

Future research should prioritize chemically well-characterized and ideally *Boswellia* preparations standardized to defined amounts of pentacyclic triterpenes, comprehensive reporting of experimental methodologies, and well-designed preclinical and clinical studies. In particular, well-designed clinical trials evaluating standardized frankincense-based topical formulations are warranted to determine their efficacy, safety, optimal dosage, and therapeutic positioning in psoriasis management. Such studies are required to determine whether *Boswellia*-derived topical preparations may have a future role in the management of chronic inflammatory skin diseases.
